# Which Foods May Be Addictive? The Roles of Processing, Fat Content, and Glycemic Load

**DOI:** 10.1371/journal.pone.0117959

**Published:** 2015-02-18

**Authors:** Erica M. Schulte, Nicole M. Avena, Ashley N. Gearhardt

**Affiliations:** 1 Department of Psychology, University of Michigan, Ann Arbor, Michigan, United States of America; 2 Department of Medicine, New York Obesity Research Center, Mount Sinai- St. Luke’s Hospital, New York, New York, United States of America; Colorado State University, UNITED STATES

## Abstract

**Objectives:**

We propose that highly processed foods share pharmacokinetic properties (e.g. concentrated dose, rapid rate of absorption) with drugs of abuse, due to the addition of fat and/or refined carbohydrates and the rapid rate the refined carbohydrates are absorbed into the system, indicated by glycemic load (GL). The current study provides preliminary evidence for the foods and food attributes implicated in addictive-like eating.

**Design:**

Cross-sectional.

**Setting:**

University (Study One) and community (Study Two).

**Participants:**

120 undergraduates participated in Study One and 384 participants recruited through Amazon MTurk participated in Study Two.

**Measurements:**

In Study One, participants (*n* = 120) completed the Yale Food Addiction Scale (YFAS) followed by a forced-choice task to indicate which foods, out of 35 foods varying in nutritional composition, were most associated with addictive-like eating behaviors. Using the same 35 foods, Study Two utilized hierarchical linear modeling to investigate which food attributes (e.g., fat grams) were related to addictive-like eating behavior (at level one) and explored the influence of individual differences for this association (at level two).

**Results:**

In Study One, processed foods, higher in fat and GL, were most frequently associated with addictive-like eating behaviors. In Study Two, processing was a large, positive predictor for whether a food was associated with problematic, addictive-like eating behaviors. BMI and YFAS symptom count were small-to-moderate, positive predictors for this association. In a separate model, fat and GL were large, positive predictors of problematic food ratings. YFAS symptom count was a small, positive predictor of the relationship between GL and food ratings.

**Conclusion:**

The current study provides preliminary evidence that not all foods are equally implicated in addictive-like eating behavior, and highly processed foods, which may share characteristics with drugs of abuse (e.g. high dose, rapid rate of absorption) appear to be particularly associated with “food addiction.”

## Introduction

The prevalence of obesity in the United States continues to increase, with more than 85% of adults projected to be overweight or obese by 2030 [1]. Health care costs associated with obesity currently comprise almost 10% of national health care expenditures [2] and are projected to increase to 15% over the next 15 years [1]. There has been little success at preventing excessive weight gain or developing weight loss treatments that are effective long-term [3]. Multiple causes contribute to the obesity epidemic, such as increased energy intake, increased availability and ease of access to foods, larger portion sizes, and decreased physical activity [4–6]. Although the causes of obesity are multifactorial, one potential contributing factor is the idea certain foods may be capable of triggering an addictive response in some individuals, which may lead to unintended overeating.

Gearhardt et al. [7] developed and validated the Yale Food Addiction Scale (YFAS), which uses DSM-IV criteria for substance dependence to quantify symptoms of addictive-like eating (see Table 1). “Food addiction” is characterized by symptoms such as loss of control over consumption, continued use despite negative consequences, and an inability to cut down despite the desire to do so [8]. Addictive-like eating has been associated with increased impulsivity and emotional reactivity, which are similarly implicated in substance-use disorders [9]. Thus, “food addiction” may share common behavioral attributes with other addictive disorders. Neuroimaging studies have also revealed biological similarities in patterns of reward-related dysfunction between “food addicts” and substance-dependent individuals. Individuals endorsing symptoms of “food addiction” exhibit increased activation in reward-related regions (e.g., striatum, medial orbitofrontal cortex) in response to food cues, consistent with other addictive disorders [10]. Further, higher scores on the YFAS have been associated with a composite genetic index of dopamine signaling [11]. This multilocus genetic profile has been related to dopamine signaling capacity, which may also be a risk factor for addictive disorders [12,13].

**Table 1:** Endorsement of YFAS Symptoms in Study One and Two.

YFAS Symptom, Based on DSM-IV Criteria for Substance Dependence	Study One (% of participants endorsing symptom)	Study Two (% of participants endorsing symptom)
(1) Substance taken in larger amount and for longer period than intended	11.7%	15.1%
(2) Persistent desire or repeated unsuccessful attempt to quit	91.7%	91.7%
(3) Much time/activity to obtain, use, recover	23.3%	25.8%
(4) Important social, occupational, or recreational activities given up or reduced	7.5%	20.6%
(5) Use continues despite knowledge of adverse consequences (e.g. failure to fulfill role obligation, use when physically hazardous	15%	26%
(6) Tolerance (marked increase in amount; marked decrease in effect)	23.3%	33.9%
(7) Characteristic withdrawal symptoms; substance taken to relieve withdrawal	12.5%	24.5%
“Food addiction” diagnosis (Three or more symptoms plus clinically significant impairment or distress)	6.7%	10.2%

Much like the term “drug,” which can encompass both addictive (e.g. heroin) and non-addictive (e.g. aspirin) compounds, the term “food” is also broad and refers not only to foods in their natural state (e.g. vegetables), but also those with added amounts of fat and/or refined carbohydrates (e.g. cake) or artificial sweeteners (e.g. diet soda). The term “food addiction” can be further refined because it is highly unlikely that all foods may be addictive. Identification of the specific foods or food attributes associated with this type of pathological eating is essential to an addiction framework. An addiction perspective posits a “person x substance” effect, where an individual’s predisposition for addiction interacts with an addictive agent to result in problematic use [14]. Without exposure to an addictive substance, a person vulnerable to problematic use would not develop an addiction [15]. Thus, while evidence suggests that there may be biological and behavioral overlaps between “food addiction” and substance-use disorders [16,17], a logical next step is to examine which specific foods or food attributes may be capable of triggering an addictive response.

Addictive substances are rarely in their natural state, but have been altered or processed in a manner that increases their abuse potential. For example, grapes are processed into wine and poppies are refined into opium. A similar process may be occurring within our food supply. There are naturally occurring foods that contain sugar (e.g., fruits) or foods that naturally contain fat (e.g., nuts). Notably, sugar (or refined carbohydrates) and fat rarely occur in the same food naturally, but many palatable foods have been processed to have artificially elevated quantities of both (e.g. cake, pizza, chocolate). Further, in our modern food environment, there has been a steep increase in the availability of what is often referred to as “highly processed foods”, or foods that are engineered in a way that increases the amount of refined carbohydrates (i.e., sugar, white flour) and/or fat in the food [18]. Although cooking or stirring is a form of processing, the current study utilizes the term “highly processed” to refer to foods that have been designed to be particularly rewarding through the addition of fat and/or refined carbohydrates. Foods that have other added ingredients, like fiber or vitamins, would not be considered “highly processed” by the current definition, unless the food also had added levels of fat and/or refined carbohydrates. It is plausible that like drugs of abuse, these highly processed foods may be more likely to trigger addictive-like biological and behavioral responses due to their unnaturally high levels of reward.

In substance-use disorders, one result of processing addictive substances is often a higher concentration of the addictive agent [19]. An increased potency, or concentrated dose, of an addictive agent increases the abuse potential of the substance. For example, water has little, if any, abuse potential, whereas beer (which contains on average 5% ethanol) is more likely to be abused. In contrast, hard liquor contains a higher dose of ethanol (between 20–75%) and is more likely to be related to problematic use than beer [20]. Similarly, the addition of fat and refined carbohydrates (like sugar) into highly processed foods may increase the “dose” of these ingredients, beyond what one might find in a natural food (such as in fruit or nuts). Increasing the “dose” of these ingredients may elevate the abuse potential of these foods in a manner akin to traditionally addictive substances.

Additionally, addictive substances are altered to increase the rate at which the addictive agent is absorbed into the bloodstream. For example, when a coca leaf is chewed, it is considered to have little addictive potential [21]. However, once it is processed into a concentrated dose with rapid delivery into the system, it becomes cocaine, which is highly addictive [22]. Similarly, highly processed foods, compared to naturally occurring foods, are more likely to induce a blood sugar spike. This is important, because there is a known link between glucose levels and activation of areas of the brain that are involved with addiction [23]. While a food’s glycemic load (GL) and glycemic index (GI) are both measures of the blood sugar spike [24–26], the current study utilizes GL because it is calculated using not only the magnitude of the blood sugar spike but also the dose (grams) of refined carbohydrates. Many foods with a high GL (e.g. cake, pizza) have been highly processed to increase the concentration of refined carbohydrates, such as white flour and sugar. Simultaneously, fiber, protein, and water are stripped from the food, which further increases the rate that the refined carbohydrates are absorbed into the system. For example, the sugar in a highly processed, high GL food, such as a milk chocolate bar, will be more quickly absorbed into the system than the natural sugars in a banana (low GL). This is because the banana is unprocessed, and though it contains sugar, it also has fiber, protein, and water, which slow the rate that the sugar enters the bloodstream. Given our knowledge of addictive substances, it may then be hypothesized that the chocolate would have a higher abuse potential than the banana. In summary, it appears that highly processed foods may be altered in a manner similar to addictive substances to increase the food’s potency (dose) and absorption rate [27].

Although there is little evidence in humans of what foods may be addictive, animal models suggest that highly processed foods are associated with addictive-like eating. Rats with a propensity towards binge eating exhibit addictive-like behavior in response to highly processed foods, such as Oreo Double Stuf cookies or frosting, but not to their typical chow [28,29]. Rats maintained on a diet of highly processed foods, such as cheesecake, exhibit downregulation in the dopamine system that also occurs in response to drugs of abuse [30]. Further, rats are motivated to seek out highly processed foods despite negative consequences (foot shock), which is another feature of an addiction [31]. Therefore, at least in animal models, overconsumption of highly processed foods, but not standard rat chow, appears to produce some addictive-like characteristics. This reinforces the idea that not all foods are likely to be equally associated with addictive-like eating behaviors.

Animal research has also investigated whether food attributes typically added to highly processed foods, such as sugar and fat, are particularly implicated in “food addiction.” In animals, it appears that sugar may be most associated with addictive-like eating [32]. Rats given intermittent access to sugar in their diet exhibit a number of behavioral indicators of addiction, such as binge consumption, tolerance, and cross-sensitization to other drugs of abuse [33]. When the sugar is removed from the diet or when an opiate antagonist is administered, rats experience signs of opiate-like withdrawal, such anxiety, teeth chattering, and aggression [33–35]. Sugar bingeing has been shown to increase mu-opioid receptor binding [36] in a similar manner to drugs of abuse [37,38]. Bingeing on sucrose produces a repeated increase of dopamine, rather than the gradual decline over time, which is a hallmark of addictive substances [39,40]. Thus, behavioral and biological evidence in animal models suggest that sugar may be an addictive agent in highly palatable foods.

However, rats bingeing on sugar do not experience an increase in body weight [38]. Thus, fat may also be an important food attribute for addictive-like eating, but through different mechanisms. Bingeing on fat-rich foods (e.g. shortening) is associated with an increase in body weight but may not result in opiate-like withdrawal symptoms [39]. One explanation is that fat may alter effects on the opioid system or enhance the palatability of the food [38,39]. Interestingly, when rats binge on highly processed foods high in both sugar and fat, they experience changes in the dopamine system akin to drugs of abuse but do not exhibit signs of opiate-like withdrawal [32]. This suggests that sugar and fat may both play important, yet distinct, roles in the addictive potential of highly processed foods.

Little is known about how these food characteristics might result in addictive-like eating in humans. Given the findings in animals, highly processed foods may be more likely to be consumed in an addictive manner. For drugs of abuse, processing may increase a substance’s addictive potential (e.g. processing grapes into wine) by elevating the dose, or concentration, of the addictive agent and expediting its rate of absorption into the bloodstream. Applying this logic to food attributes, it may follow that refined carbohydrates (e.g., sugar, white flour) and fat are important contributors to addictive-like eating. However, it is not just the presence of these nutrients, as they also appear in naturally occurring foods. Rather, the addictive potential of a food is likely to increase if the food is highly processed to increase the amount, or dose, of fat and/or refined carbohydrates and if the refined carbohydrates are absorbed into the bloodstream quickly (high GL). An essential next step in the consideration of “food addiction” is to determine which foods or food attributes pose the greatest risk in the development of addictive-like eating behaviors in humans.

The initial part of the current study is the first to systematically examine which foods and food attributes are most implicated in “food addiction”. Specifically, participants complete the YFAS, which examines behavioral indicators of addictive-like eating and are then asked to identify which foods they are most likely to experience problems with, as described in the YFAS, out of a set of 35 foods varying in levels of processing, fat, and GL. These nutritional attributes of interest were selected based on the addiction literature and the pharmacokinetic properties (e.g. dose, rate of absorption) of drugs of abuse. This approach allows us to rank the 35 foods from most to least associated with addictive-like eating behaviors based on participants’ responses. Additionally, the second part of current study examines which food attributes are implicated in addictive-like eating by examining a food’s level of processing, GL, and amount of fat. We also utilize hierarchical linear modeling to investigate whether food attributes (e.g. amount of fat) are more related to addictive-like eating behavior for certain individuals. Specifically, we explore whether gender, body mass index (BMI), and the endorsement of symptoms on the YFAS alter the association between food attributes and addictive-like eating. For example, BMI may be associated with greater craving for foods high in fat and salt, such as bacon and chips [41]. Thus, different food attributes may be more or less relevant to addictive-like eating based on the characteristics of the participant. In summary, the current study addresses an existing gap in the literature by examining which foods or food attributes are implicated in “food addiction” and explores whether certain food attributes are particularly relevant based on gender, BMI, and endorsement of addictive-like eating behaviors.

## Study One

### Methods

Ethics Statement

The University of Michigan Health and Behavioral Sciences Institutional Review Board approved the current study (HUM00082154) and written informed consent was obtained from all participants.

Participants

Participants included 120 undergraduates, who were recruited from flyers on campus or through the University of Michigan Introductory Psychology Subject Pool. Participants recruited through flyers were compensated ($20) and individuals recruited through the Introductory Psychology Subject Pool received course credit for their time. Participants were aged 18 to 23 (mean = 19.27 years, *SD* = 1.27), 67.5% were female, 72.5% were Caucasian, 19.2% were Asian/Pacific-Islander, 5% were Hispanic, 4.2% were African-American, and 2.4% were Other. BMI ranged from underweight to obese (mean = 23.03, *SD* = 3.20).

Procedures and Assessment Measures

Participants completed the YFAS [7], which is a 25-item self-report measure that operationalizes addictive-like eating behaviors based on the DSM-IV criteria for substance dependence. The instructions for the YFAS prime the participant to think of foods high in fat and/or refined carbohydrates when they read the phrase “certain foods” in the questions. For example, one question states, “Over time, I have found that I need to eat more and more of certain foods to get the feeling I want, such as reduced negative emotions or increased pleasure.” The current study aimed to identify which foods were most likely to be consumed in an addictive way. In order to avoid priming, we removed the language in the YFAS instructions that told individuals to think of foods high in fat and/or refined carbohydrates and replaced it with the following phrase: “When the following questions ask about “certain foods”, please think of any food you have had a problem with in the past year.”

Next, we developed a forced-choice task, where participants were provided with the following instructions: “The previous questionnaire asked about the problems people may have with certain foods. We are interested in which foods may be most problematic for you. In the following task, you will be presented with food items. Please choose the food item that you are more likely to experience problems with. An example of what we mean by ‘problems’ is having trouble cutting down on the food or losing control over how much of the food you eat. An example of what we do not mean by ‘problems’ is feeling like you aren’t eating enough of the food.” Participants were then presented with two food pictures at a time, out of a bank of 35 total foods, and selected which one they were more likely to experience “problems” with, as described by the YFAS. Food pictures were accompanied with text describing the item (e.g. cookie), and if certain foods were commonly consumed in multiple ways that could markedly change their nutritional information, indicators were used to specify the type of food presentation being examined. For example, cucumbers are commonly consumed with vegetable dips containing added fat. Thus, we specified that we were interested in the likelihood of experiencing problematic eating behaviors with cucumbers not accompanied by dip. Each food was compared to all other foods by the end of the forced choice task. Next, participants reported demographic information (ethnicity, gender, year in school, and age) and last, height and weight were measured.

Food Stimulus Set

The foods were systematically selected to have varying levels of processing (18 foods were categorized as “highly processed”, marked by the addition of fat and/or refined carbohydrate content (e.g. cake, chocolate, pizza, chips), 17 foods were categorized as “not processed” (e.g. banana, carrots, nuts), fat (M = 8.57g, SD = 9.18, range = 0–30), sodium (M = 196.57mg, SD = 233.97, range = 0–885), sugar (M = 7.40g, SD = 9.82, range = 0–33), carbohydrates (M = 20.74g, SD = 16.09, range = 0–56), GL (M = 10.31, SD = 9.07, range = 0–29), fiber (M = 1.69g, SD = 2.39, range = 0–10), protein (M = 7.89g, SD = 11.12, range = 0–43), and net carbohydrates (e.g. grams of carbohydrates minus grams of fiber) (M = 19.09g, SD = 15.06, range = 0–49). The correlations between the main nutritional attributes of interest were: processing/fat, r = 0.314, *p* > 0.05; processing/GL, r = 0.756, *p* < 0.01; and fat/GL, r = 0.239, *p* > 0.05. Due to the high correlation between processing and GL, we did not simultaneously include them in any statistical model. Food items fit into roughly four categories: 1) high in both fat and refined carbohydrates/sugar (e.g. chocolate, French fries), 2) high in fat but not refined carbohydrates/sugar (e.g. cheese, bacon), 3) high in refined carbohydrates/sugar but not fat (e.g. pretzels, soda), or 4) low in both fat and refined carbohydrates/sugar (e.g. broccoli, chicken). Nutrition facts were gathered from www.nutritiondata.com or food company websites and based on standard portion size. Pictures were acquired from digitally available sources of food pictures and were presented during the task using E-Prime 2.0 software [42]. Food items were displayed in color on a white background and were of equal size.

Data Analytic Plan

For each food item, the outcome was the frequency for which that food was selected as being more problematic, as described by the YFAS, than other foods. Since each food item was compared to all other foods in the task, the maximum number of times a food could have been reported as problematic was 34. Thus, the more problematic a food was reported to be, the higher the likelihood that food’s frequency count approached or reached 34.

### Results and Discussion

YFAS symptoms ranged from 0 to 6 (mean = 1.85, *SD* = 1.33). Table 1 shows the frequency for which each YFAS symptom was endorsed. YFAS symptom count was associated with BMI (r = 0.211, *p* = 0.020), but not gender. Although there was a significant association of YFAS symptom count with BMI, the association was not large enough to raise concerns about multicollinearity. Table 2 provides the average frequency count and rank order of the 35 food items. Level of processing appeared to be the most influential attribute for whether a food was associated with problematic, addictive-like eating behaviors. For example, the top ten foods chosen most frequently during the task were highly processed, with added amounts of fat and refined carbohydrates/sugar (e.g. chocolate, pizza, cake). Further, thirteen unprocessed foods make up the bottom of the list, meaning these foods were least associated with problems described in the YFAS.

**Table 2:** Study One: Average frequency count of how often a food was selected as problematic.^1^

Rank	Food	Frequency	Processed?	GL	Fat (grams)	Sodium (milligrams)
1	Chocolate	27.60	Y	14	13	35
2	Ice Cream	27.02	Y	14	15	98
3	French Fries	26.94	Y	21	19	266
4	Pizza	26.73	Y	22	10	551
5	Cookie	26.72	Y	7	4	63
6	Chips	25.38	Y	12	10	160
7	Cake	24.84	Y	24	10	260
8	Popcorn (Buttered)	23.39	Y	26	30	771
9	Cheeseburger	21.26	Y	17	28	885
10	Muffin	20.81	Y	29	19	380
11	Breakfast Cereal	20.61	Y	22	6	270
12	Gummy Candy	20.58	Y	22	0	15
13	Fried Chicken	20.18	Y	7	26	441
14	Soda (Not Diet)	20.07	Y	16	0	15
15	Rolls (Plain)	20.01	Y	15	1	450
16	Cheese	19.36	N	0	9	174
17	Pretzels	19.20	Y	15	1	380
18	Bacon	18.05	N	0	12	647
19	Crackers (Plain)	16.88	Y	11	6	223
20	Nuts	16.43	N	3	13	179
21	Steak	16.16	N	0	24	38
22	Granola Bar	14.39	Y	10	6	160
23	Eggs	13.93	N	0	7	94
24	Chicken Breast	12.61	N	0	5	104
25	Strawberries	12.42	N	1	0	2
26	Apple	10.21	N	4	0	2
27	Corn (No Butter or Salt)	9.92	N	8	1	6
28	Salmon	9.44	N	0	22	109
29	Banana	9.34	N	12	0	1
30	Carrots (Plain)	9.08	N	2	0	66
31	Brown Rice (Plain, No Sauce)	8.79	N	20	2	2
32	Water	6.91	N	0	0	0
33	Cucumber (No Dip)	6.83	N	0	0	1
34	Broccoli	6.48	N	0	0	30
35	Beans (No Sauce)	6.47	N	7	1	380

^1^ Information in parentheses was explicitly stated to participants. This decision was made because the addition of these ingredients would change the processing categorization and nutrition information of that food item.

As hypothesized, highly processed foods (with added fat and/or refined carbohydrates) appeared to be most associated with behavioral indicators of addictive-like eating. To explore this further, Study Two examined which foods are implicated in addictive-like eating in a more representative, diverse sample. Additionally, we utilized an outcome variable that enabled us to employ hierarchical linear modeling [43] and explore whether individual differences moderate which food attributes were reported as problematic and linked to behavioral indicators of addictive-like eating.

## Study Two

### Methods

Ethics Statement

The University of Michigan Health and Behavioral Sciences Institutional Review Board approved the current study (HUM00089084) and written informed consent was obtained from all participants.

Participants

A total of 398 participants were recruited using Amazon’s Mechanical Turk (MTurk) worker pool to complete a study about eating behaviors and were paid ($0.40) for their time, which is comparable compensation for other studies using MTurk [44]. Paolacci and Chandler [44] observed that although MTurk’s worker pool is not nationally representative, it is diverse and can replace or supplement traditional convenience samples. Individuals were excluded from analysis if they reported information outside of possible bounds (*n* = 1) (e.g. weight of 900 pounds), for reporting age outside of our defined 18–65 range (*n* = 8), for omitting gender (*n* = 3) or for incorrectly answering “catch questions” (*n* = 2), which attempted to identify individuals providing answers without reading the question items. Participants (*n* = 384) were aged 18 to 64 (mean = 31.14, *SD* = 9.61), 59.4% were male, 76.8% were Caucasian, 12% were Asian or Pacific Islander, 8.9% were African-American, 6.5% were Hispanic, and 2.8% were Other. BMI, as calculated by self-reported of height and weight, ranged from underweight to obese (mean = 26.95, *SD* = 6.21) and YFAS symptoms ranged from 0 to 7 (mean = 2.38, *SD* = 1.73). Table 1 shows the frequency for which each YFAS symptom was endorsed. YFAS symptom count was associated with BMI (r = 0.217, *p* < 0.001) but not gender.

Procedures and Assessment Measures

Participants completed the aforementioned version of the YFAS, which did not include food-priming information, and were presented with instructions for an adapted version of the forced-choice task in Study One. Rather than comparing each food against one another, participants were asked to rate how likely they were to experience problems, as described by the YFAS, with each of the 35 foods on a Likert scale from 1 (not at all problematic) to 7 (extremely problematic). Demographic information (ethnicity, gender, income, and age) and self-reported height and weight were also collected.

Data Analytic Plan

Hierarchical linear modeling with robust standard errors [43] was used to analyze the relationship between nutritional characteristics of the foods and the food ratings. A two-level regression analysis was conducted, consisting of participants’ ratings of the 35 foods at level one, nested within 384 participants at level two. This analytic approach allowed us to evaluate 1) the influences of food-specific characteristics on the rating representing the likelihood that the food was associated with behavioral indicators of addictive-like eating (at level one) and 2) the idiographic influences of participant-specific characteristics on the relationship between food-specific characteristics and food ratings (at level two).

### Results

Table 3 provides the mean rating assigned to each food item in ranked order. Food items with higher ratings were reported as more problematic, as indicated by addictive-like eating behaviors described in the YFAS. Consistent with Study One, highly processed foods, or foods with added amounts of fat and/or refined carbohydrates, were most associated with addictive-like eating behaviors. Nine out of the ten foods at the top of the list were highly processed and high in both fat and refined carbohydrates. Soda (not diet) was the exception, which is highly processed and high in refined carbohydrates, but not fat.

**Table 3:** Study Two: Average food ratings based on 7-point Likert scale (1 = not problematic at all, 7 = extremely problematic).^1^

Rank	Food	Mean Rating	Processed?	GL	Fat (grams)	Sodium (milligrams)
1	Pizza	4.01	Y	22	10	551
2	Chocolate	3.73	Y	14	13	35
2	Chips	3.73	Y	12	10	160
4	Cookie	3.71	Y	7	4	63
5	Ice Cream	3.68	Y	14	15	98
6	French Fries	3.60	Y	21	18	266
7	Cheeseburger	3.51	Y	17	28	885
8	Soda (Not Diet)	3.29	Y	16	0	15
9	Cake	3.26	Y	24	10	260
10	Cheese	3.22	N	0	9	174
11	Bacon	3.03	N	0	12	647
12	Fried Chicken	2.97	Y	7	26	441
13	Rolls (Plain)	2.73	Y	15	1	450
14	Popcorn (Buttered)	2.64	Y	26	30	771
15	Breakfast Cereal	2.59	Y	22	6	270
16	Gummy Candy	2.57	Y	22	0	15
17	Steak	2.54	N	0	24	38
18	Muffin	2.50	Y	29	19	380
19	Nuts	2.47	N	3	13	179
20	Eggs	2.18	N	0	7	94
21	Chicken Breast	2.16	N	0	5	104
22	Pretzels	2.13	Y	15	1	380
23	Crackers (Plain)	2.07	Y	11	6	223
24	Water	1.94	N	0	0	0
25	Granola Bar	1.93	Y	10	6	160
26	Strawberries	1.88	N	1	0	2
27	Corn (No Butter or Salt)	1.87	N	8	1	6
28	Salmon	1.84	N	0	22	109
29	Banana	1.77	N	12	0	1
30	Broccoli	1.74	N	0	0	30
30	Brown Rice (Plain, No Sauce)	1.74	N	20	2	2
32	Apple	1.66	N	4	0	2
33	Beans (No Sauce)	1.63	N	7	1	2
34	Carrots	1.60	N	2	0	66
35	Cucumber (No Dip)	1.53	N	0	0	1

^1^ Information in parentheses was explicitly stated to participants. This decision was made because the addition of these ingredients would change the processing categorization and nutrition information of that food item.

Food Ratings and Processing

In the level-one equation, the dummy-coded variable of processing (highly processed and unprocessed) was specified as a main effect for each participant’s food ratings.

Level-One Equation for Processing as a Predictor of a Food’s Rating:

$$RATINGS=\;\beta_{0}+\;\beta_{1}\;*(PROCESSI)+r$$

The intercept for the level-one equation (*β*_0_) is interpretable as the model-predicted food rating when the processing variable is zero, which indicates an unprocessed food. In this case, the model predicts a rating of 2.147 for an unprocessed food. The partial slope (*β*_0_) indicates the impact that level of processing has on a food’s rating. In this level-one model, the value of 0.689 for *β*_1_ would indicate that a food’s rating increases by 0.689 points for a highly processed, relative to unprocessed, food.

Chi-square tests revealed significant variation across participants in the intercept and utilization parameter (processing) at level one, *χ* ^*2*^(383) = 2172.10 and 598.72 respectively, *p* <0.001. This means that participant-specific characteristics had an effect on the association between a food’s processing level and food ratings. Thus, level two analyses were conducted and both parameters were treated as random effects.

Level-two equations explored whether participant-specific predictors of variability emerged for the two random level-one parameters. Participant-specific predictors of BMI (centered), YFAS symptom count (centered), and gender (dummy-coded) were examined. Intercepts in the level-two equations (*γ*_00_ and *γ*_10_) are interpreted as the average value of each level-one parameter for a participant with mean values (or zero if dummy coded) on all level-two predictors. For example, *γ*_10_ signifies the average impact of processing on food ratings for a male (gender = 0) participant of average BMI and symptom count. Further, the partial slopes in each level-two equation measure the impact of processing on food ratings associated with a one-unit increase in the level-two participant-specific predictor. For example, *γ*_12_ is interpreted as the change in the impact of processing that occurs for every additional symptom endorsed on the YFAS, holding other level-two predictors at their mean values.

Level-Two Equations for Participant-Specific Predictors of Level-One Parameters

$$\beta_{0}=\;\gamma_{00}+\;\gamma_{01}*(BMI)+\;\gamma_{02}*(SYMPTOMC)+\;\gamma_{03}*(GENDER)+\;\mu_{0}$$

$$\beta_{1}=\;\gamma_{10}+\gamma_{11}*(BMI)+\;\gamma_{12}*(SYMPTOMC)+\gamma_{13}*(GENDER)+\;\mu_{1}$$

The average food rating *γ*_00_ was 2.241; the average participant rated unprocessed foods an average of 2.241 on the Likert scale from 1 to 7. Examination of the intercepts for the utilization parameter suggested a significant effect of processing on the average participant’s food ratings. Effect sizes were calculated using procedures recommended by Oishi and colleagues [45]. Processing was a large, positive predictor for the degree in which a food was reported as problematic and associated with addictive-like eating behaviors (*γ*_10_ = 0.653, d = 1.444, *p* < 0.001). The average participant’s food rating for a highly processed food was 0.653 points higher than the rating for an unprocessed food. In other words, the average participant reported a rating of 2.241 for unprocessed foods and a rating of 2.894 for highly processed foods (2.241 + 0.653). Thus, the model suggests that participants reported more behavioral indicators of addictive-like eating with highly processed foods.

YFAS symptom count was a moderate-to-large, positive predictor for problematic food ratings of unprocessed foods, when controlling for BMI and gender (*γ*_01_ = 0.157, d = 0.536, *p* < 0.001). Gender also emerged as a small, positive predictor for whether an unprocessed food was reported as problematic, with men reporting more problems with unprocessed foods than women (*γ*_03_ = -0.233, d = 0.236, *p* < 0.022). Two participant-specific predictors of variability emerged for the level-one parameter of processing. BMI was a small, positive predictor for the food ratings of highly processed foods when controlling for the effects of YFAS symptomology and gender (*γ*_12_ = 0.012, d = 0.235, *p* = 0.023); increases in BMI were associated with elevated problematic food ratings for highly processed foods. Additionally, YFAS symptom count emerged as a small-to-moderate, positive predictor for the effect of processing on food ratings when controlling for BMI and gender (*γ*_11_ = 0.063, d = 0.324, *p* = 0.002); each one unit increase in symptom count was associated with a 0.063 increase in a highly processed food’s rating. Thus, when reporting food ratings of addictive-like eating problems, level of processing was particularly important for individuals with elevated BMI and symptoms of addictive-like eating. Finally, gender was not significantly associated with the level-one parameter of processing.

Food Ratings, Fat, and GL

Next, we examined which additional food attributes increase the likelihood of experiencing problems with a certain food, as specified by the YFAS. In order to mitigate multicollinearity and obtain more information about what food characteristics may be most strongly associated with addictive-like eating, we ran a second model that did not include processing. Based on addiction literature, this second model specified fat and GL as food attributes of interest, as both may have potential implications for dose and rate of absorption. Specifically, highly processed foods increase the dose (or amount) of fat and/or refined carbohydrates. Further, GL captures not only the dose of refined carbohydrates, but also the rate in which they are absorbed in the system. Thus, these food attributes appear to capture potential pharmacokinetic similarities between highly processed foods and drugs of abuse.

The level-one equation indicated two main effects on participants’ food ratings of problematic, addictive-like eating behavior: fat (centered) and GL (centered). The intercept for the level-one equation (*β*_0_) reflects the model-predicted food rating for a food with average fat grams and average GL. The partial slopes (*β*_1_ and *β*_2_) are interpreted as the impact of fat and GL, respectively, on food ratings.

Level-One Equation for Fat and GL as a Predictor of a Food’s Rating

$$RATINGS=\;\beta_{0}+\beta_{1}*(FAT)+\beta_{2}*(GL)+r$$

Chi-square tests revealed significant variation across participants’ ratings of foods that vary in GL, *χ* ^*2*^ (383) = 524.218, *p* < 0.001, but not fat grams (*χ* ^*2*^ (383) = 404.791, *p* = 0.213). Therefore, only participant-specific predictors of the intercept and GL were examined. All three parameters were treated as random effects. The same level-two predictors (i.e., YFAS symptoms, BMI, gender) were entered into this model to examine the change in impact of GL on food ratings based on participant-specific characteristics.

Level-Two Equations for Participant-Specific Predictors of Level-One Parameters

$$\beta_{0}=\;\gamma_{00}+\;\gamma_{01}*(BMI)+\;\gamma_{02}*(SYMPTOMC)+\;\gamma_{03}*(GENDER)+\;\mu_{0}$$

$$\beta_{1}=\;\gamma_{10}+\mu_{1}$$

$$\beta_{2}=\;\gamma_{20}+\;\gamma_{21}*(BMI)+\;\gamma_{22}*(SYMPTOMC)+\;\gamma_{23}*(GENDER)+\;\mu_{2}$$

A participant with mean values (or zero if dummy coded) on the level-two parameters reported an average rating of 2.62 for a food item with average fat and GL values (*γ*_00_). Fat content was found to be a large, positive predictor of a food’s rating (*γ*_10_ = 0.025, d = 1.581, *p* < 0.001), meaning that a food’s rating of addictive-like eating problems increased by 0.025 for each one-unit increase in fat grams from the average value. In other words, foods with elevated fat content were reported to be related to addictive-like eating problems. Though sodium has been proposed as another important contributor to addictive-like eating, multicollinearity between sodium and fat prevent these variables from being placed in the same model (r = .623, *p* < 0.001). We assessed fat and sodium independently, and though both were significant level-one predictors, we determined that fat had a larger effect size than sodium (fat: d = 1.853, *p* < 0.001; sodium: d = 1.223, *p* < 0.001). Thus, fat was utilized in the second model.

GL was also a large, positive predictor of food ratings (*γ*_20_ = 0.021, d = 0.923, *p* < 0.001), indicating that a food’s rating of problematic eating behavior increased by 0.021 for each one-unit increase in GL from the average. Further, we found that GL had a significantly larger effect size than either sugar or net carbohydrates when put into our second model with fat (GL: d = 0.923; sugar: d = 0.814; net carbohydrates: d = 0.657). Thus, GL which captures both the amount of refined carbohydrates and how rapidly they are absorbed by the system, appears to be particularly associated with problematic eating, as defined by the YFAS.

YFAS symptom count was a large, positive predictor of food ratings for a food with average fat grams and GL, controlling for the effects of BMI and gender (*γ*_01_ = 0.180, d = 0.645, *p* < 0.001) One participant-specific predictors of variability emerged for the level-one parameter of GL. YFAS symptom count was a small, positive predictor of a food’s rating based on GL when controlling for BMI and gender (*γ*_21_ = 0.003, d = 0.297, *p* = 0.004); each one unit increase in symptom-count endorsement was associated with a 0.003 increase in food rating for a food with average GL. Thus, when reporting problematic eating behavior, GL was particularly important for individuals reporting symptoms of addictive-like eating. Gender and BMI were not significantly associated with the rating of foods associated with GL.

Summary

In summary, level of processing emerged as a large, positive predictor of food ratings of problematic, addictive-like eating behavior. YFAS symptomology and gender (male) were predictors for whether an individual reported problems with an unprocessed food. Further, YFAS symptom count and BMI both emerged as positive predictors for the association between highly processed foods and ratings of problematic eating behavior, as indicated by the YFAS. Thus, individuals with elevated BMI and/or symptoms of addictive-like eating were more likely to report experiencing addictive-like behaviors to highly processed foods. Additionally, fat and GL were significant predictors of problematic food ratings. YFAS symptom count emerged as a positive predictor for food ratings for the “average” food with mean grams of fat and GL values. Finally, GL was particularly predictive of problematic food ratings for individuals with an elevated YFAS symptom count, meaning that individuals endorsing addictive-like eating behaviors were especially likely to report problems with high GL foods.

## Discussion

Though evidence of “food addiction” continues to grow, no previous studies have yet examined which foods or food attributes are likely implicated in addictive-like eating. The identification of a potentially addictive profile in certain foods is important for furthering our understanding of the “food addiction” construct and for informing public health education and food policy initiatives [46–48].

In a sample of undergraduates, we observed that highly processed foods with added levels of fat and/or refined carbohydrates (like white flour and sugar), were most likely to be associated with addictive-like eating behaviors. Additionally, we hypothesized that a food’s fat grams and GL may also be predictive, based on the pharmacokinetics of addictive substances (e.g. dose, rapid rate of absorption). This was examined using a more diverse participant sample in Study Two, which indeed found processing, fat, and GL to be predictive of whether a food was associated with problematic, addictive-like eating behavior, as described by the YFAS. Further, individuals with elevated BMI and/or YFAS symptom count reported greater difficulties with highly processed foods, and men indicated that unprocessed foods (e.g., steak, nuts, cheese) were more problematic than women. Though addictive-like eaters reported more problems generally, high GL was particularly indicative of whether a food was associated with addictive-like eating behaviors for participants endorsing symptoms of “food addiction.” No individual differences were significantly predictive of the relationship between amount of fat and whether a food was related to problematic, addictive-like eating.

### Food-Specific Characteristics

Processing

Processing appears to be an essential distinguishing factor for whether a food is associated with behavioral indicators of addictive-like eating. Highly processed foods are altered to be particularly rewarding through the addition of fats and/or refined carbohydrates (like white flour and sugar). While cooking or stirring is a form of processing, foods that have been cooked or stirred but do not contain added fat and/or refined carbohydrates (e.g. steak) are not categorized as highly processed in the current study. The present findings support and extend the preclinical literature [7,49,50] by demonstrating that all foods are not equally implicated in addictive-like eating, and highly processed foods, which do not occur in nature, appear to be most problematic, as described by the YFAS. Thus, it appears that an unprocessed food, such as an apple, is less likely to trigger an addictive-like response than a highly processed food, such as a cookie. The finding that processing was the most predictive factor for whether a food was associated with addictive-like eating behaviors is preliminary evidence for narrowing the scope of which foods are implicated in the construct of “food addiction.” Future research is needed to determine whether “food addiction” may be more appropriately titled “highly processed food addiction.”

Glycemic Load (GL)

Though level of processing was a large, positive predictor for whether a food may be likely implicated in addictive-like eating, it was necessary to examine which food attributes associated with highly processed foods are related to addictive-like eating problems. A food’s GL reflects not only the amount of refined carbohydrates in a food, but also the rate in which they are absorbed into the system. Similarly, it is well known that with addictive substances, a concentrated dose of an addictive agent and its rapid rate of absorption increases the addictive potential. Previous research has suggested that foods with higher GL may be capable of activating reward-related neural circuitry (e.g. striatum), akin to addictive substances, and increasing craving and hunger, which may lead to overeating [23,24,51,52]. Thus, we hypothesized that a food’s GL, a measure of the blood sugar spike after consumption, would be predictive of addictive-like eating. We observed that GL was a large, positive predictor for whether a food was reported as problematic, specified by the YFAS. Further, we found that GL was more predictive than sugar or net carbohydrate content for problems related to addictive-like eating. Thus, it appears that it is not just the quantity of refined carbohydrates (like white flour and sugar) in a food, but the rapid speed in which they are absorbed into the system that is the most significant predictor of whether a particular food is associated with behavioral indicators of addictive-like eating.

Fat

We also hypothesized that the amount of fat grams would be important in predicting whether a food was associated with problems related to addictive-like eating. Previous studies indicate that fat may enhance palatability in the mouth and activate somatosensory brain regions [53,54]. In the current study, we found that higher fat content was a large, significant predictor of problematic, addictive-like eating. Further, it appears that greater amounts of fat may increase the likelihood that a food will be consumed problematically regardless of individual differences and not uniquely for those who report consuming food in an addictive-like way.

### Individual Difference Factors

YFAS

YFAS symptoms were associated with ratings of problems related to addictive-like eating for unprocessed foods and for foods with average fat content and GL. Thus, individuals with elevated YFAS scores may generally experience more problematic eating behavior than individuals who do not report consuming food in an addictive-like manner. YFAS symptom count was also a small-to-moderate, positive predictor for the relationship between problematic food ratings and processing. In other words, individuals endorsing symptoms of addictive-like eating were especially likely to report problems, as indicated by the YFAS, with highly processed foods, which is consistent with the hypothesis that these foods may have a greater addictive potential.

YFAS symptomology was also linked to an increased association between GL and problematic food ratings. In other words, individuals endorsing symptoms of addictive-like eating reported increased difficulty with foods containing rapidly absorbed refined carbohydrates, which produce a large blood sugar spike. This reinforces the shared importance of rate of absorption in potentially addictive foods and drugs of abuse. Interestingly, problematic consumption of foods with a high glycemic index (GI), another measure of the blood sugar spike that is related to GL, has been linked to the development of new-onset substance-use disorders in post-surgical bariatric patients, and high-GI foods may activate reward-related brain regions (e.g. nucleus accumbens, striatum) after consumption [23,55]. This provides further evidence for the role of GL and the blood sugar spike in the experience of a potentially addictive response to certain foods.

Endorsement of addictive-like eating behavior was not associated with the relationship between fat content and problematic food ratings. It may be that individuals generally report problematic consumption of high-fat foods, but fat is less predictive of whether someone actually experiences an addictive-like process in response to a certain food. This is supported by animal models demonstrating that opiate-like withdrawal, a marker of an addictive process, is observed in response to sucrose being removed from the diet but not fat [32]. In the current study, it appears that the amount of fat predicts whether a food is reported as problematic, regardless of individual differences, but is not strongly associated with the endorsement of addictive-like eating behavior. This suggests that fat may be related to a general tendency to overeat, which may have public health implications for the prevention and treatment of problematic eating. Additionally, many highly processed foods with added fats often also contain added refined carbohydrates (e.g. chocolate, French fries). Thus, additional research is warranted to disentangle the unique predictive power of fat and refined carbohydrates/GL.

BMI and Gender

BMI was a small, positive predictor for whether a highly processed food was associated with problematic, addictive-like eating. This suggests that processing may not only increase a food’s “addictive potential,” but also play a role in the obesity epidemic. Elevated BMI was not related to the relationship of fat or GL with food ratings. The current study found that men reported more problems with unprocessed foods (e.g., steak, cheese) than women, which suggests that men may experience problematic eating behavior with a wider range of foods.

### Limitations

The current study had some limitations. First, the data for Study Two was collected using Amazon MTurk. While the participant sample was more representative than Study One’s undergraduate population, it may not be considered a nationally representative sample [56] and replication may increase generalizability. Similarly, since the current studies examined college students and adults, the findings may not be applicable to non-college students or youth. Additionally, the range of food ranks was limited. Foods that were reported as most problematic had mean ratings of just greater than 4, meaning no food was ranked on average as extremely problematic (a score of 7). Intuitively, this makes sense, since our sample ranged from individuals reporting no addictive-like eating symptoms to those meeting diagnostic criteria for “food addiction.” It is expected that some individuals would not experience addictive-like eating symptoms to any foods. Future studies may consider label magnitude scaling [57]. Compared to Likert scales, label magnitude scaling approaches attempt to address individual differences in perceived severity of problematic eating that may differ by level of pathology. Finally, we did not collect observational data to assess the frequency that these foods were consumed, which is an important next step in this research. It is also unknown whether the context of consumption (e.g., snack, meal, binge episode) may influence whether a food is associated with behavioral indicators of addictive-like eating. Thus, the current findings are limited to participants’ reports of whether certain foods are perceived to be associated with addictive-like eating behavior. Finally, height and weight were self-reported in Study Two, which may lead to inaccuracy. While several studies have found that self-reported height and weight are highly correlated with direct measurements [58,59], additional research may consider utilizing direct measurement.

### Conclusions

In summary, the current study found that highly processed foods, with added amounts of fat and/or refined carbohydrates (e.g., sugar, white flour), were most likely to be associated with behavioral indicators of addictive-like eating. Additionally, foods with high GL were especially related to addictive-like eating problems for individuals endorsing elevated symptoms of “food addiction.” Individuals endorsing symptoms of addictive-like eating behavior may be more susceptible to the large blood sugar spike of high GL foods, which is consistent with the importance of dose and rate of absorption in the addictive potential of drugs of abuse. Collectively, the findings provide preliminary evidence for the foods and food attributes implicated in “food addiction” and for proposed parallels between pharmacokinetic properties of drugs of abuse and highly processed foods. As an important next step in the evaluation of “food addiction,” future studies should also expand on the current findings by measuring biological responses and directly observing eating behaviors associated with highly processed foods in order to examine whether addictive-like mechanisms, such as withdrawal and tolerance, may be present.
